# A novel miR-0308-3p revealed by miRNA-seq of HBV-positive hepatocellular carcinoma suppresses cell proliferation and promotes G1/S arrest by targeting double CDK6/Cyclin D1 genes

**DOI:** 10.1186/s13578-020-00382-7

**Published:** 2020-02-27

**Authors:** Xiaoming Dai, Ruixue Huang, Sai Hu, Yao Zhou, Xiaoya Sun, Pucheng Gui, Zijian Yu, Pingkun Zhou

**Affiliations:** 1 The First Affiliated Hospital, University of South China, 69 Chuanshan Road, Hengyang, 421001 Hunan People’s Republic of China; 2 Department of Occupational and Environmental Health, Xiangya School of Public Health, Central South University, Changsha, 410078 China; 3 Institute for Chemical Carcinogenesis, State Key Laboratory of Respiratory, Guangzhou Medical University, Guangzhou, 511436 People’s Republic of China; 4 Beijing Key Laboratory for Radiobiology, Beijing Institute of Radiation Medicine, 27 Taiping Road, Haidian District, Beijing, 100850 People’s Republic of China

**Keywords:** miRNA, HBV, HCC, Carcinogenesis

## Abstract

**Background:**

Persistent infection with hepatitis B virus (HBV) accounts for the majority of hepatocellular carcinoma (HCC), but the molecular mechanisms underlying liver carcinogenesis are still not completely understood. Increasing evidence demonstrates that microRNAs (miRNAs) play significant functional roles in virus–host interactions. The aim of this study was to explore differentially expressed miRNA profiles and investigate the molecular mechanism of miR-0308-3p in HBV-positive HCC carcinogenesis.

**Methods:**

High-throughput sequencing was used to detect novel miRNAs in three samples of HBV-positive HCC tissue compared to matched HBV-negative HCC tissue. The Cancer Genome Atlas (TCGA) database was used to mine miRNAs related to HBV-positive HCC. Bioinformatics analyses were conducted to predict the miRNAs’ possible biological and pathway regulatory functions. Quantitative polymerase chain reaction (qPCR) was then applied to evaluate the expression levels of randomly selected miRNAs. CCK-8 was used to measure cell proliferation and cell cycles were analyzed using flow cytometry. A dual luciferase reporter gene assay was used to confirm the downstream targets of miR-0308-3p.

**Results:**

In total, there were 34 overlapping miRNAs in both our miRNA-seq data and the TCGA database. We found two overlapping miRNAs in both the HBV-positive HCC samples and the TCGA database, and 205 novel pre-miRNA sequences were predicted. miR-522 and miR-523 were markedly overexpressed in HBV-positive HCC and were associated with a significantly poorer long-term prognosis (miR-522, HR 2.19, 95% CI 1.33–3.6, *p* = 0.0015; miR-523HR 1.5, 95% CI 1–2.44, *p* = 0.0047). Of note, we found that the novel miR-0308-3p was markedly downregulated in HBV-positive HCC samples and HCC cancer cell lines compared with HBV-negative HCC samples and adjacent normal hepatic tissue. Moreover, elevated expression of miR-0308-3p was found to inhibit proliferation of cancer cells by promoting G1/S cell cycle arrest but did not influence the apoptosis of cancer cells. A dual luciferase reporter activity assay identified that miR-0308-3p acted directly on the target sequence of the CDK6 and Cyclin D1 mRNA 3ʹUTR to suppress CDK6 and Cyclin D1 expression.

**Conclusions:**

MiR-0308-3p upregulation dramatically suppressed HCC cell proliferation and induced G1/S cell cycle arrest by directly targeting CDK6/Cyclin D1. These findings reveal a novel molecular mechanism for activation of G1/S arrest in HCC and may prove clinically useful for developing new therapeutic targets.

## Introduction

Hepatocellular carcinoma (HCC) comprises the vast majority of primary liver cancers [1]; it is the sixth most prevalent cancer and third most frequent cause of cancer-associated deaths worldwide [2]. The incidence of HCC has risen gradually, from 1.51 cases per 100,000 in 2011 to 6.20 cases per 100,000 worldwide in 2014 [3], and this increase is anticipated to continue into the future [4]. According to the 2018 cancer statistics, there are 841,000 new cases of HCC worldwide each year, and about 55% of HCC patients are from China [5]. Notably, approximately 83% of HCC cases arise in non-Western countries, with China accounting for slightly more than 50% of all new diagnoses in 2012. There are numerous established HCC risk factors, of which chronic hepatitis infection, in particular hepatitis B virus (HBV) infection, and subsequent liver fibrosis and cirrhosis are the most critical [6, 7]. It is estimated that approximately 2 billion people have been infected with HBV worldwide. Generally, HBV contributes to the development of HCC through integration of HBV DNA into the host genome, which induces both genomic instability and direct insertional mutagenesis of diverse cancer-related genes [8]. Globally, approximately 240 million people are chronically infected with HBV, and approximately 25% of those affected eventually develop HCC. Moreover, approximately 60% of HCC cases in Africa and Asia are associated with HBV infection [9]. Despite its prevalence, the mechanism for HBV-positive HCC is still poorly understood. A better understanding of the molecular mechanisms underlying HBV-positive HCC carcinogenesis would help in the development of more effective molecular-targeted interventions for the primary prevention and treatment of HCC.

MicroRNAs (miRNAs) are small non-coding RNAs that participate in post-transcriptional regulation of gene expression [10]. Accumulating evidence implies that miRNAs play essential roles in the development and progression of HCC, and they are attracting increasing attention as a possible new class of key HCC biomarkers [11]. Current research regarding the use of miRNAs as biomarkers for HCC can be categorized into three strategies. One is to use high-throughput sequencing to construct miRNA expression profiles for HCC tissue and adjacent normal tissue [12]. The second is to mine currently available HCC-related RNA data in online RNA-seq databases, including The Cancer Genome Atlas (TCGA) and NCBI Gene Expression Omnibus (GEO) databases [11]. The third is to use classical qRT-PCR assays to detect miRNAs in clinical HCC patient samples [13]. These three strategies are commonly used to investigate key miRNAs, which may lead to the diagnosis and treatment of HCC at an earlier stage. miR-139 was recently investigated as a new biomarker for chronic HBV-positive HCC using microarrays [14]. Similarly, Chen et al. detected expression of a number of miRNAs previously associated with lung cancer in HCC patients’ serum using qRT-PCR and discovered that miR-331-3p may be a new diagnostic and prognostic marker for HCC [15]. Lin et al. conducted bioinformatics analyses using NCBI GEO and found a number of new miRNAs that were predicted to be associated with HCC development [11]. Zhang et al. investigated miRNA profiles in 328 HCC patient samples from the TCGA database and generated an HCC-specific 7-miRNA signature that was validated as an independent prognostic biomarker [16]. Liang L conducted bioinformatics analyses using both the GEO and TCGA databases, subsequently validating the previously reported miR-338-5p as a biomarker for diagnosing HCC, as well as discovering 423 of its potential target genes [17]. Although rapid advances in RNA sequencing have led to an exponential increase in the prediction of miRNAs [18–21], and an enormous number of their downstream target genes, most of these have been generated from a single analysis strategy rather than integration of HCC clinical sequencing, HCC-related RNA-seq database analysis, and classical qRT-PCR. Furthermore, functional analyses regarding most newly discovered miRNAs in HBV-positive HCC samples are limited to gene ontology (GO) analysis, Kyoto Encyclopedia of Genes and Genomes (KEGG) pathway analysis, or survival analysis, and further investigation of their molecular mechanisms through cell experimentation is seldom conducted. Li et al. integrated microarray and TCGA database mining to investigate the role of miR-200b in renal cell carcinoma carcinogenesis and demonstrated its previously undescribed role as a suppressor of tumor metastasis by directly destabilizing Laminin subunit alpha 4 (LAMA4 mRNA) [22]. Hence, we hypothesized that integration of high-throughput sequencing of clinical HBV-positive HCC samples and RNA-seq database mining would lead to the discovery of more biomarkers and a better understanding of the molecular-mechanisms in HBV-positive HCC carcinogenesis.

## Methods and materials

### Patient information, TCGA introduction, cell lines and plasmids construction and tranfection

In total, 15 pairs of fresh specimens containing human HBV-positive and HBV-negative HCC tissue were collected from the First Affiliated Hospital of the University of South China. The baseline information of the enrolled patients is shown in Table 1. All the enrolled HCC patients’ diagnoses were based on case histories, clinical symptoms, predictors, and/or tissue biopsy according to the National Diagnosis Criteria. After the samples were collected, they were sent to RiboBio Co., Ltd. (Guangzhou, China) for immediate high-throughput sequencing analysis. This study was performed in accordance with the Declaration of Helsinki and was approved by the institutional review board (IRB) of the First Affiliated Hospital of the University of South China (IRB approval no. NHUH-2017-38). All enrolled patients signed informed-consent forms.

**Table 1:** Clinical and pathological characteristics of 15 pairs of HBV-positive and HBV-negative HCC tissue

Variable	Number	Percentage (%)	Variable	Number	Percentage (%)
Age (years)			Tumor necrosis		
< 45	5	33.3	No	6	40
≥ 45	10	66.7	Yes	9	60
Sex			Perineural invasion		
Male	12	80	Yes	3	20
Female	3	20	No	12	80
Occupation			Tumor invasion	6	15
Farmer	7	46.7	Yes	7	46.7
Teacher	3	20	No	8	53.3
Worker	5	33.3	Vascular invasion		
Histological grade			Yes	6	40
I	2	13.3	No	9	60
II	7	46.7			
III	5	33.3			
IV	1	6.7			
Tumor size (cm)	4.59 ± 3.78				

The HCC-related miRNA-seq and RNA-seq data were downloaded from the TCGA database available at http://portal.gdc.cancer.gov/. The sequencing data were all publicly available, therefore no ethical issues were involved [23].

The human HepG2 and SMMC-7721 cell lines were originally purchased from ATCC. Both cell lines were maintained in Dulbecco’s modified Eagle medium (DMEM; GIBCO BRL, Grand Island, NY) supplemented with 10% FBS and 1% PS (100 units/ml penicillin, 100 μg/ml streptomycin) and incubated at 37 °C with 5% CO_2_.

The CDK6 and Cyclin D1 without 3ʹ-UTR was synthesized by Beijing Genomics Institute (Beijing, China) and subcloned into the pEX-3 vector (GenePharma, Shanghai, China) to generate the miR-0308-3p-resistant pEX-3-CDK6 vector and Cyclin D1 following the manufacturer’s instructions. An empty pEX-3 vector was used as a control. miR-0308-3p-resistant pEX-3-CDK6 vector, miR-0308-3p-resistant pEX-3- Cyclin D1 vector and control plasmid were transfected using Lipofectamine 3000 Reagent based on the agent-box instruction.

### High-throughput sequencing

High-throughput sequencing was conducted using an Illumina HiSeq 2500 (Illumina, Inc., San Diego, CA, USA) followed by DNA library construction. For construction of the DNA library, first total RNA was isolated from HCC samples with or without HBV infection using an miRNA isolation kit (Invitrogen, Carlsbad, CA, USA) according to the manufacturer’s instructions. Then, RNA molecules 18–30 nt in size were separated by gel electrophoresis and 3ʹ and 5ʹ adapters were ligated to the RNAs. The ligation products were constructed by reverse transcription polymerase chain reaction (RT-PCR), and PCR products 140–160 bp in size were enriched to generate a cDNA library [24]. After sequencing, the reads were filtered to achieve clean tags. All the clean tags were aligned with small RNAs using miRBase version 21 (http://www.mirbase.org), Rfam12.1 (http://rfam.xfam.org), and piRNAbank (http://pirnabank.ibab.ac.in) to identify known miRNAs. For novel miRNA candidate prediction, the following selection criteria were applied using Mireap v0.2 software: removal of isomers, hairpin structure, stable secondary structure with binding free energy less than − 20 kcal/mol, and located at an intergenic spacer.

### Quantitative reverse-transcription polymerase chain reaction (qRT-PCR)

For analysis of miRNA expression, qRT-PCR was used. Briefly, total RNA was isolated from HCC samples using an miRNA isolation kit (Invitrogen, Carlsbad, CA, USA) following the manufacturer’s instructions. Then, 1 µg of total RNA was reverse-transcribed. SYBR Premix Ex Taq II (Takara, Tokyo, Japan) was used for RT-qPCR analysis, according to the manufacturer’s instructions. The relative expression of mRNA or miRNA was calculated using the 2^−ΔΔCt^ method and normalized to the expression of GAPDH or U6, respectively. All PCRs were performed in triplicate. The miRNA primer sets were purchased from GenePharma (Suzhou, China). The primer pairs of the relevant genes are shown in Additional file 1: Table S1.

### Western blotting analysis, flow cytometry analysis, and apoptosis detection

Western blotting was used to evaluate levels of protein expression according to our previously published studies [25–27]. The cell-cycle distribution of HepG2 was estimated using flow cytometry analysis. HepG2 and SMMC-7721 cells were placed in 12-well plates at a density of 2 × 10^5^ cells/well and transfected with or without an miR-0308-3p mimic followed by 24 h incubation. Dimethyl sulfoxide (DMSO) was used as a control. For estimation of DNA content, the cells were washed in PBS and fixed in ethanol at – 20 °C. This was followed by re-suspension in PBS with 40 μg/ml PI and RNase A (0.1 mg/ml) and Triton X-100 (0.1%) for 30 min in a dark room at 37 °C [28].

For the detection of apoptotic cell populations, HepG2 and SMMC-7721 cells at a density of 2 × 10^5^ cells/well were seeded in six-well plates and treated with or without an miR-0308-3p mimic for 24 h. The cells were then stained with 4ʹ-6-diamidino-2-phenylindole (DAPI) and imaged under a fluorescence microscope. Annexin V/IP was used to estimate apoptotic cell populations.

### CCK-8 assay

For the HepG2 cell-proliferation analysis, HepG2 cells were collected at passage 3–4 and inoculated in six-well plates at a density of 4 × 10^6^ cells/well. The effects of miR-0308 on cell viability were detected using a standard cell-counting kit-8 (CCK-8) according to the manufacturer’s instructions. The optical density (OD) of the cells in each group was tested by measuring the absorbance at 570 nm using a microplate reader [29].

### miRNA target prediction, Gene Ontology (GO) analysis, and Kyoto Encyclopedia of Genes and Genomes (KEGG) pathway analysis

miRNA target prediction was conducted using miRDB (http://mirdb.org/). For target prediction of known miRNAs, the miRNA name was entered directly as the Target search, while for target prediction of novel miRNAs, the RNA sequence was entered in the Custom Prediction search.

The Gene Ontology (GO) database is a consortium-based dataset that offers information on gene products regarding cellular components, molecular function, and biological processes using ontologies. There are approximately 500,000 annotations about Homo sapiens in the database. Predicted targets for a miRNA are evaluated as a group using GO terms. Significant GO categories are identified using statistics for gene-function enrichment [30].

KEGG was developed by Kanehisa Laboratories in 1995 and is now a widely available bioinformatics database of the high-level functions and utilities of biological systems from the perspective of genomes and molecules (https://www.genome.jp/kegg/). The current version consists of 18 databases, including KEGG Pathway, KEGG Genome, and KEGG Drug. In this study, we performed GO and KEGG analyses on the significantly differentially expressed miRNAs, novel miRNAs, and miRNAs with overlapping expression patterns.

### Dual luciferase reporter assay

The wild-type CDK6 mRNA 3ʹ-untranslated region (3ʹUTR) (Genbank: NM001145306.2), Cyclin D1 3'UTR (Genbank:BC023620.2)  and the mutant sequences at the predicted target sites for miR-0308-3p in the CDK6 mRNA 3ʹUTR were cloned into the pmirGLO vector to generate the pmirGLO-CDK6_3ʹUTR_wt, pmirGLO-Cyclin D1_3'UTR_wt, pmirGLO-CDK6_3ʹUTR-Mut and pmirGLO-Cyclin D1_3'UTR_Mut constructs, respectively. HepG2 cells were seeded onto 24-well plates (6.0 × 10^4^ cells/well) for 24 h before co-transfection with 1 μg of the reporter plasmids and 1 μg of the pmirGLO internal control plasmids. After 8 h, the medium was replaced and the cells were transfected with 100 nM miR-0308 mimic or control (DMSO). After incubation for 48 h, the transfected cells were lysed and their luciferase activity was detected using the Dual-Luciferase Reporter Assay System (Cat. No. E1910; Promega, Madison, WI, USA). Firefly luciferase activity was normalized to that of Renilla luciferase and each group was detected in triplicate according to previous reports [29, 31].

### Survival assay

A Kaplan–Meier survival analysis was performed on the available Kaplan–Meier plotter website (http://www.kmplot.com/analysis/), which is capable of assessing the effects of 54,000 genes on survival in 21 types of cancer, including HCC [32]. The following five miRNAs with overlapping expression patterns were used in the survival analysis: miR-523, miR-517a, miR-372, miR-522, and miR-524-5p.

### Statistical analyses

Statistical analyses were performed using SPSS software (ver. 19.0; IBM Corp., Armonk, NY, USA). Quantitative data were expressed as mean ± standard deviation. A one-way analysis of variance (ANOVA) followed by the least significant difference post-hoc test and t-test were used for the comparison of means. We considered *p*-values < 0.05 to be statistically significant.

## Results

### HCC patients with HBV-positive infection showed global changes in miRNA expression

**Fig. 1:**
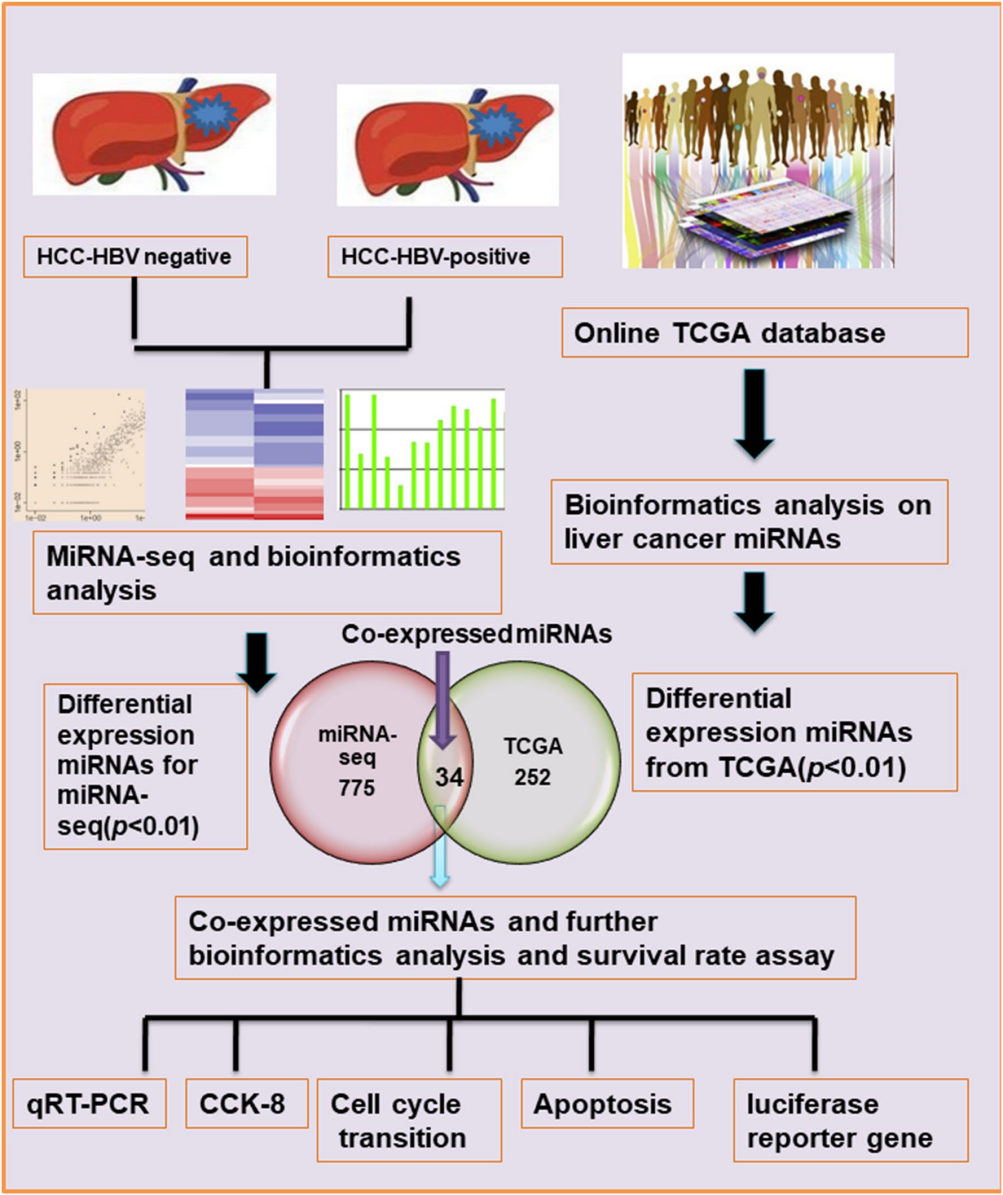
A flow chart of the experimental design. First, to explore the newly identified miRNAs in HBV-positive HCC patients, five pairs of HCC and adjacent matched non-HCC tissue from HBV-positive and HBV-negative patients were collected using high-throughput sequencing. Bioinformatics analyses were performed to predict the biological function of significant differentially expressed miRNAs. Second, the online TCGA database was mined for miRNAs related to HBV-positive HCC, and a bioinformatics analysis of these miRNAs was also performed. Third, differentially expressed miRNAs with overlapping expressionpatterns in both our miRNA-seq data and the TCGA database were chosen for further bioinformatics analysis. First, to validate the high-throughput sequencing results, a few novel miRNAs and miRNAs with overlapping expressionpatterns were randomly chosen for qRT-PCR analysis in 15 pairs of HCC samples with or without HBV infection. Meanwhile, a survival analysis was conducted using a few miRNAs with overlapping expressionpatterns from the TCGA database. Finally, functional experiments were performed, including CCK-8, cell cycle transition, apoptosis, and luciferase reporter assays to further uncover the miRNA’s underlying mechanisms in HBV-positive HCC

**Fig. 2:**
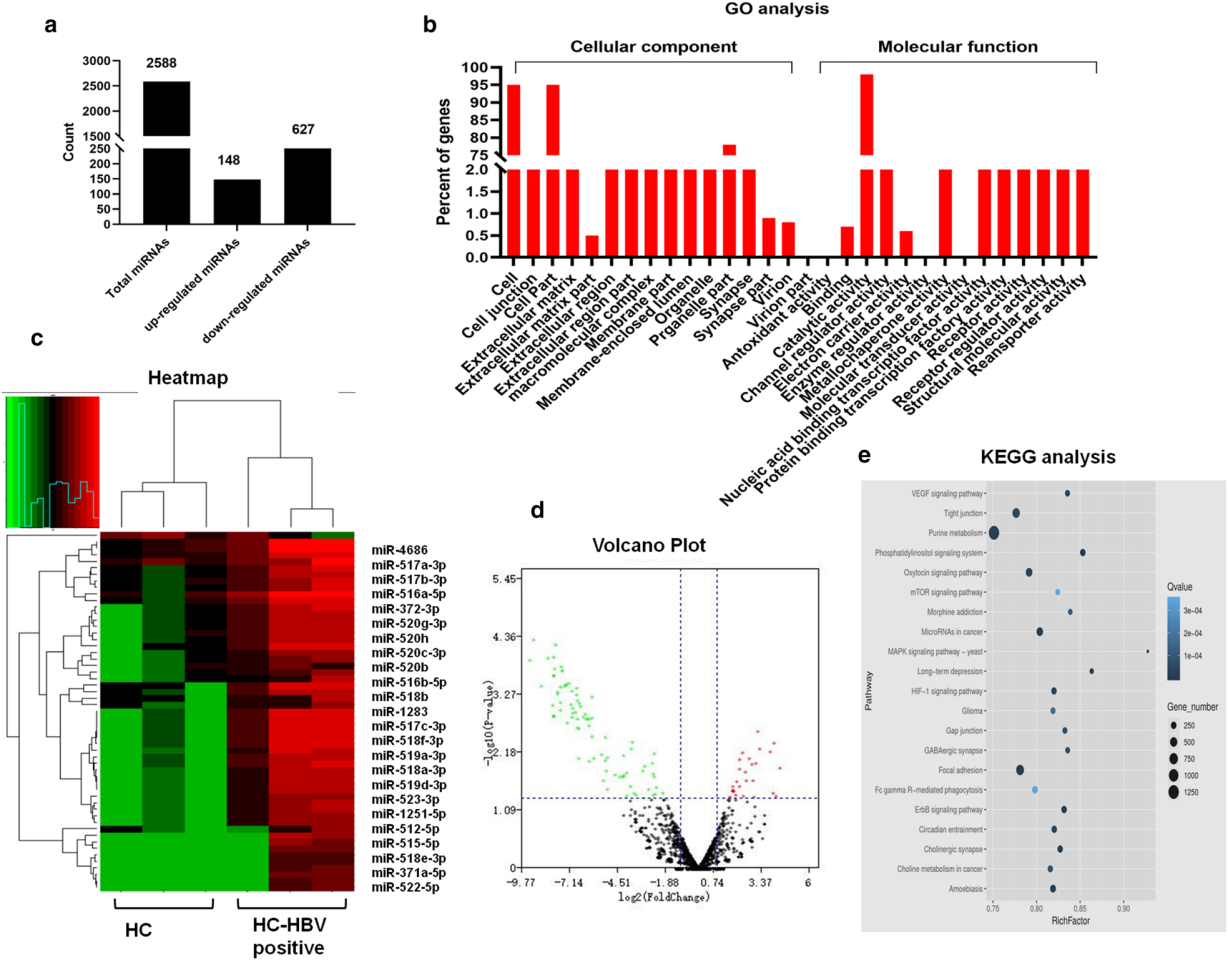
Analysis of differentially expressed miRNAs in HCC patients with HBV-negative infection and HBV-positive infection using high-throughput sequencing. **a** The total, upregulated, and downregulated differentially expressed miRNAs. **b** GO analysis of the differentially expressed miRNAs. The y-axis represents the number of genes and the x-axis represents the GO terms. **c** Hierarchical clustering (heatmap) indicating the differences in miRNA expression profiling between the two groups. Green and red represent the downregulated and upregulated miRNAs, respectively. **d** Volcano plot showing the expression profiling in the two groups. The vertical green lines refer to a 2.0-fold (log2 scaled) upregulation and downregulation, respectively. The horizontal green line corresponds to a p-value of 0.05 (− log10 scaled). The red points in the plot represent differentially expressed miRNAs with statistical significance. **e** KEGG analysis of the differentially expressed miRNAs

Figure 1 indicates the study design. To compare miRNA expression profiles between HCC patients with or without HBV infection and to mine possible biomarkers for HCC patients with HBV-positive infection, five HBV-positive HCC samples, five HBV-negative HCC samples, and the adjacent non-tumor tissue were used. In total, 2588 miRNAs were identified, of which 775 were differentially expressed with a fold-change of more than 1. Figure 2a shows the 148 upregulated and 627 downregulated miRNAs in the HBV-positive HCC samples, and Table 2 shows the top 20 upregulated and downregulated miRNAs from Fig. 2. The most highly upregulated miRNA was miR-4686 with an approximately sixfold change (*p* < 0.01). Three miRNAs—miR-190a-3p, miR-4662b, and miR-2681-3phad a fold-change above four. Among the downregulated miRNAs, miR-520d-3p and miR-372-3p exhibited the highest fold-changes in the HBV-positive HCC samples (*p* < 0.01).

**Table 2:** Top 20 differentially expressed miRNAs in the liver cancer patients with HBV infection

Differentially expressed miRNAs					
Up-regulated miRNAs			Down-regulated miRNAs		
miRNA	Fold	*p* value	miRNA	Fold	*p* value
hsa-miR-4686	5.998	3.54E−06	hsa-miR-520d-3p	− 9.7702	2.35E−05
hsa-miR-190a-3p	4.3576	0.012801	hsa-miR-520d-5p	− 9.1468	4.81E−05
hsa-miR-4662b	4.1318	0.042001	hsa-miR-523-3p	− 7.9785	6.01E−05
hsa-miR-2681-3p	4.0401	0.004185	hsa-miR-526b-5p	− 8.0522	0.000103
hsa-miR-4536-3p	4.0009	0.038069	hsa-miR-520f-5p	− 8.3933	0.000106
hsa-miR-3653-3p	3.834	0.006222	hsa-miR-515-5p	− 9.3867	0.000118
hsa-miR-4500	3.5273	0.097272	hsa-miR-516a-5p	− 7.6473	0.000156
hsa-miR-6134	3.5273	0.141727	hsa-miR-524-5p	− 8.0333	0.000169
hsa-miR-4732-5p	3.5273	0.142656	hsa-miR-517a-3p	− 7.5898	0.000182
hsa-miR-6125	3.4195	0.26662	hsa-miR-517b-3p	− 7.5898	0.000183
hsa-miR-3182	3.2327	0.005509	hsa-miR-526a	− 8.0758	0.000227
hsa-miR-3163	3.2311	0.196223	hsa-miR-518d-5p	− 8.0758	0.000227
hsa-miR-522-5p	3.1985	0.202067	hsa-miR-520c-5p	− 8.0758	0.000227
hsa-miR-592	3.1103	0.002617	hsa-miR-518c-5p	− 7.1951	0.000313
hsa-miR-490-3p	3.0333	0.010412	hsa-miR-517-5p	− 7.3786	0.000327
hsa-miR-2115-5p	2.9822	0.066198	hsa-miR-518a-3p	− 7.3697	0.000344
hsa-miR-1973	2.9822	0.101527	hsa-miR-520a-3p	− 8.7703	0.000355
hsa-miR-1258	2.9112	0.044361	hsa-miR-1251a-5p	− 7.4661	0.000366
hsa-miR-517a-3p	2.8827	0.021475	hsa-miR-518b	− 7.681	0.000384
hsa-miR-7850-5p	2.8156	0.29838	hsa-miR-372-3p	− 9.7702	2.35E−05

To predict the biological functions of these differentially expressed miRNAs, a GO analysis was conducted (Fig. 2b). Differentially expressed miRNAs were predicted to have the following main functions: cell-junction, cell-part, and extracellular-matrix functions in the cellular-component category; and catalytic-activity, channel-regulator-activity, and metallochaperone-activity functions in the molecular-function category. The heatmap and volcano plot illustrated that compared with HBV-negative HCC patients, the numbers of downregulated miRNAs in HBV-positive HCC patients were higher than the numbers of upregulated miRNAs (Fig. 2c, d). We then performed a KEGG analysis (Fig. 2e), which showed that the main target pathways for the differentially expressed miRNAs were purine metabolism, oxytocin signaling, miRNAs in cancer pathways, HIF-1 signaling, and focal adhesion. Taken together, the GO and KEGG data imply that HBV-infection changes miRNA expression profiles in HCC tissue. Furthermore, it may be possible to use this profile change to predict specific inflammation and tumorigenesis changes observed in HCC tissue.

### Novel miRNA prediction in HBV-positive HCC samples

**Fig. 3:**
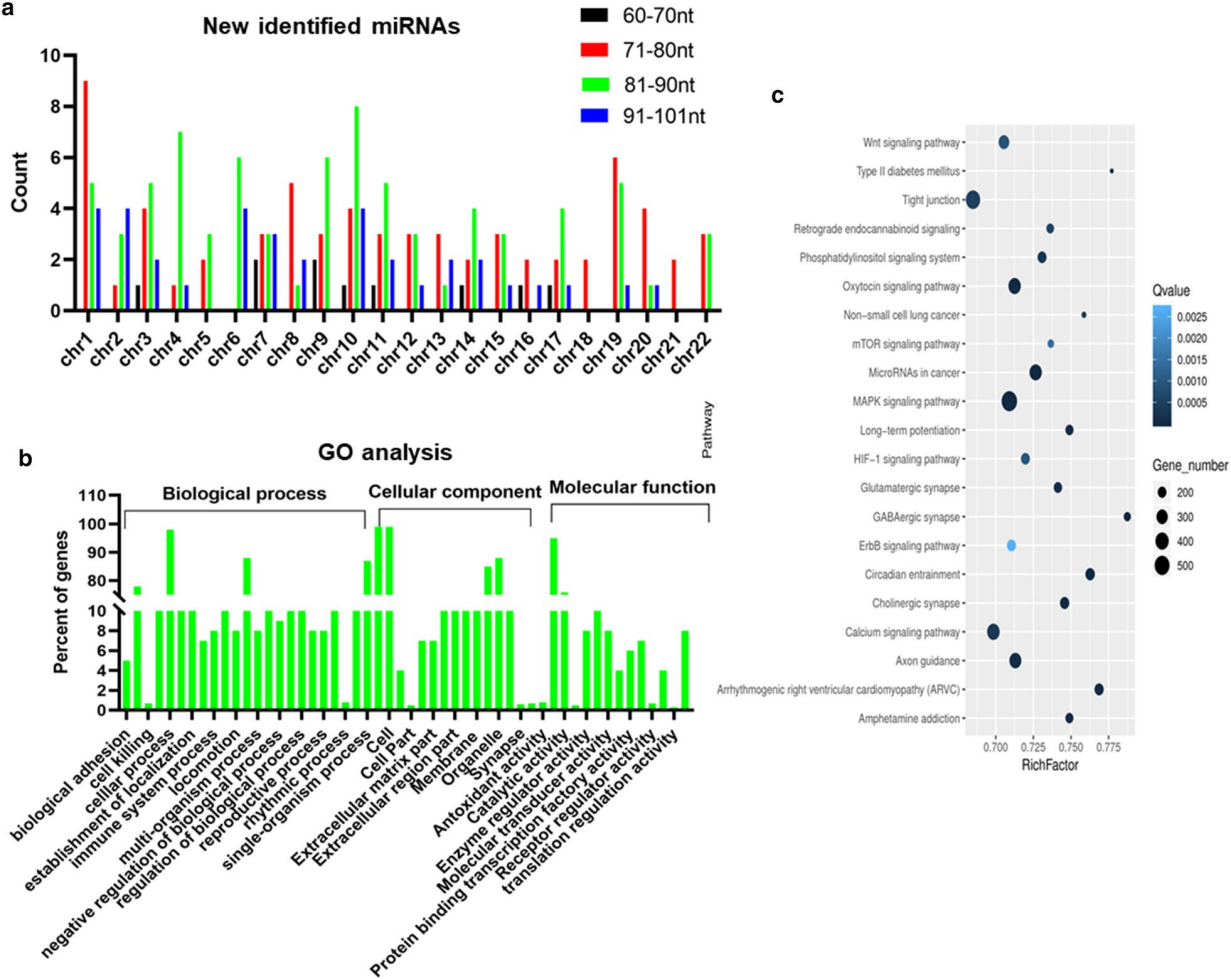
Bioinformatic analysis of novel miRNAs. **a** miRNAs were organized based on their location on human chromosomes. **b** GO analysis of the novel miRNAs. **c** KEGG analysis of the novel miRNAs

To screen for novel miRNAs, high-throughput sequencing was again used. Based on the results, most of the putative pre-miRNAs had a typical hairpin structure, with a high read depth in the mature arm and a much lower read depth in the other arm. In total, 205 novel pre-miRNA sequences were predicted, each derived from a unique genome location (Additional file 1: Table S1). Table 3 lists 25 novel miRNAs, together with the details of each sequence and read numbers. We classified the novel miRNAs based on their different chromosomal locations; novel miRNAs were found on all chromosomes except the Y chromosome (Fig. 3a). The high-throughput sequencing data showed that the length of the novel miRNAs ranged from 60 to 101 nt, and the majority were between 81 and 90 nt (Fig. 3a). The precursors of these potential novel miRNAs formed proper secondary hairpin structures with free energies ranging from − 20.60 to − 70.20 kcal/mol (Additional file 1: Table S1). Additional file 1: Table S1 also presents the probability that the predicted novel miRNAs were derived from the genomic spacer of a gene.

**Table 3:** Newly possible miRNAs identified from high-throughout sequencing

Novel-ID	Sequence	Read-number
xxx-m0034-5p	UAGCCUAUCAUACUGAUGUUAG	23
xxx-m0038-5p	UCAUACGUGGAUACCCUGGGC	10
xxx-m0043-3p	CAAGAUCGUCUGUGAACUCAGGG	18
xxx-m0087-3p	GGACCCUCAGCGGUGGAUAA	16
xxx-m0112-3p	CUGGAGUGUGGCAAUCGUGGGU	66
xxx-m0121-3p	GGCGGCGGCGGCGGCGGCGGCG	20
xxx-m0155-5p	UGGUGUGCGACGAUGGUGUGCU	17
xxx-m0189-5p	ACGGCGGUCCGCCCCCCCCCC	12
xxx-m0200-3p	UUUGAUCUGUUAGGCUUAGUUG	15
xxx-m0203-5p	UGGGCCUUCCGACUCCCAAGGCCC	10
xxx-m0207-5p	UGAGUGUGUGUGUGUGAGUAG	14
xxx-m0216-3p	CCUCCGACUCAUAGCGGGGC	22
xxx-m0218-3p	CCACUGCACUACAGGACUUGGUU	93
xxx-m0306-3p	UCGGUCCCUAACCCCCUCCGGAC	14
xxx-m0308-3p	CUGGGGUAAGCACUGCAGCUCU	15
xxx-m0345-3p	CCAGCAUUGGACUGUAGCACCA	24
xxx-m0363-3p	UCGGUCCCUAACCCCCUCCGGAC	19
xxx-m0482-5p	UGAGAGCUGGAUUCCAUGGGGC	13
xxx-m0050-3p	CAAGAUCGUCUGUGAACUCAGGG	44
xxx-m0142-3p	UUACACUAGGAUUAGAGACAAGUU	13
xxx-m0157-3p	UCUUCGUUUUAUAGUCUGAACUC	15
xxx-m0233-5p	AACACUGUGGUUAUAUCUAUAC	25
xxx-m0001-3p	ACGCGUGUCUGGGCGUUGCC	11
xxx-m0024-3p	AUAGAGAACCAUGGUCAUAGACU	102
xxx-m0025-3p	GAAGCCUUUUUCUCUGCCCA	14

To predict the biological functions of the novel miRNAs, the same strategy as for the differentially expressed miRNAs was employed. The GO analysis predicted the novel miRNAs to have the following main functions: biological-adhesion, cell-killing, and cellular process functions in the biological-process category; cellular-structure, extracellular-matrix, and cell-membrane functions in the cellular-component category; and antioxidant-activity, catalytic-activity, and enzyme-regulator-activity functions in the molecular-function category (Fig. 3b). Next, the KEGG analysis showed that the main target pathways for the novel miRNAs were Wnt signaling, oxytocin signaling, MAPK signaling, miRNAs in cancer, HIF-1 signaling, and calcium signaling (Fig. 3c).

Compared to the differentially expressed miRNAs, these novel miRNAs appeared to have exceptional functions such as cell killing and pathway-specific signaling. The data also indicated that the HBV-positive HCC samples contained more novel miRNAs compared to the HBV-negative HCC samples. The presence of these novel miRNAs may predict their involvement in the physiological regulation of HBV-positive HCC.

### Identification of miRNAs with overlapping expression patterns in both the miRNA-seq data and the TCGA database data

**Fig. 4:**
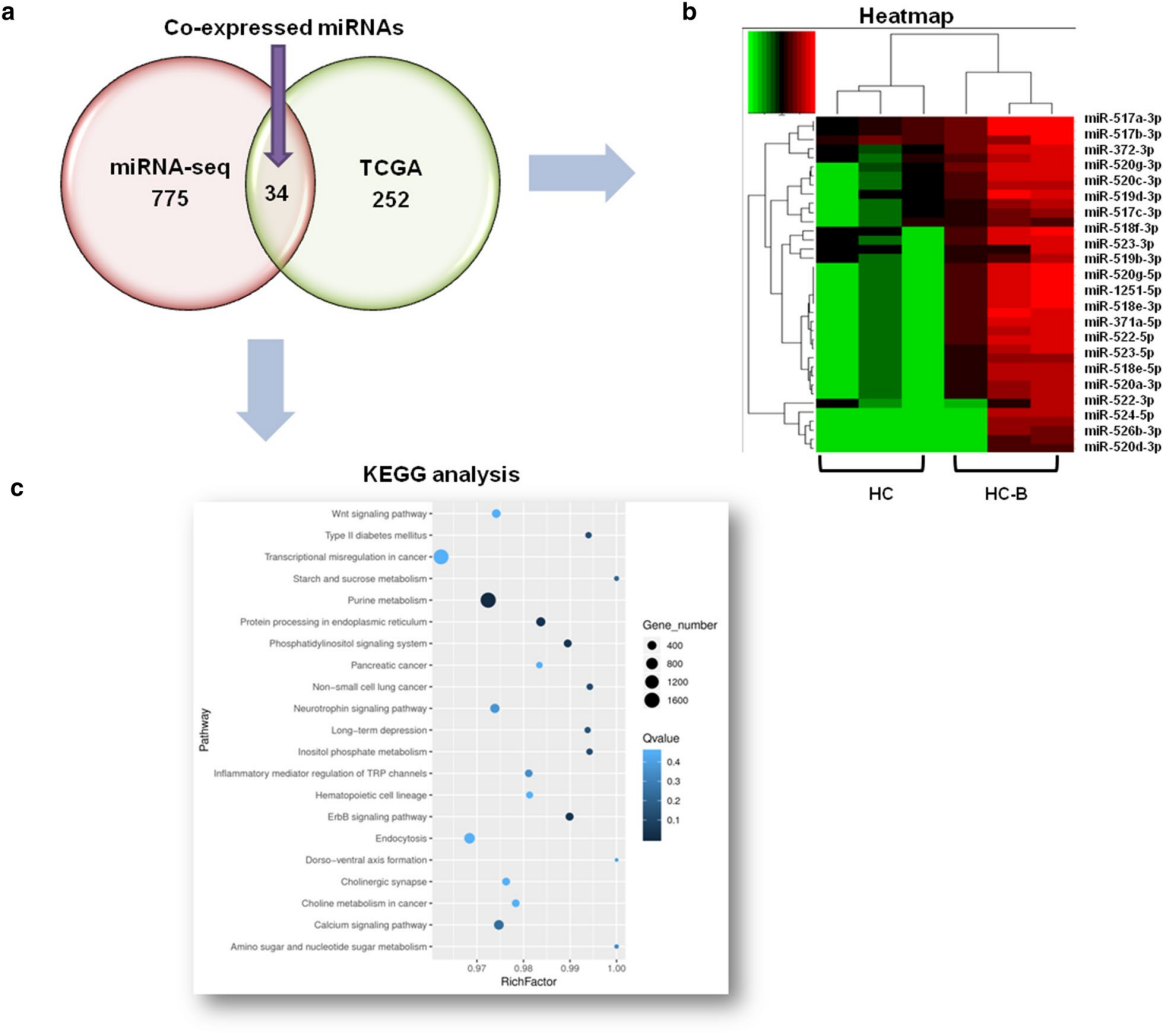
Bioinformatics analysis of overlapping-expression miRNAs in HBV-positive HCC patients picked from both our miRNA-seq data and the TCGA database. **a** A Venn diagram showing the 37 miRNAs with overlapping expression patterns in our miRNA-seq data and the TCGA database. **b** Hierarchical clustering (heatmap) indicating differences in profiling of miRNAs with overlapping expression patterns between our miRNA-seq data and the TCGA database. **c** KEGG analysis of the 37 miRNAs with overlapping expression patterns

To explore miRNAs with overlapping expression patterns, we used both our miRNA-seq data and RNAseq data from the TCGA database related to HCC with or without HBV infection. All data were obtained in accordance with relevant laws, and any necessary approvals and informed-consent documents were obtained [33]. In total, 34 miRNAs with overlapping expression patterns were identified in both the miRNA-seq data and TCGA database data (Fig. 4a). Table 4 lists the 20 miRNAs with the greatest overlap. Most of the miRNAs with overlapping expression patterns were downregulated in HBV-positive HCC samples, except for miR-520d-5p and miR-518e-5p, which were upregulated. Downregulated miR-520d-3p had the highest fold change, of 11.03, followed by downregulated miR-520d-5p and miR-520a-3p with fold changes of 8.99 and 8.42, respectively. The heatmap further demonstrated that most of these miRNAs were typically downregulated in the HBV-positive HCC samples (Fig. 4b). The miRNAs’ main target pathways were transcriptional misregulation in cancer, purine metabolism, endocytosis calcium signaling pathway, and hematopoietic cell lineages (Fig. 4c). The overlapping miRNAs were predicted to play specific roles in inflammation and carcinogenesis in HBV-positive HCC.

**Table 4:** Top 20 overlapping differential expressed miRNAs occurring in both of miRNAseq and TCGA

Novel ID	log FC	log CPM	p value	FDR significant	Up and down
hsa-miR-520d-3p	11.0341	3.783871	3.12E−05	0.0167215	Down
hsa-miR-520d-5p	8.99718	1.811335	7.07E−05	0.0209125	Down
hsa-miR-517b-3p	7.45664	7.342619	0.000141	0.0209125	Up
hsa-miR-524-3p	7.93198	0.83429	0.000143	0.0209125	Down
hsa-miR-517a-3p	7.45664	7.342619	0.000146	0.0209125	Down
hsa-miR-523-3p	7.80761	5.792401	0.000175	0.0209125	Down
hsa-miR-520c-3p	7.3379	4.781157	0.000264	0.0212975	Down
hsa-miR-526b-5p	7.76614	6.113595	0.000355	0.0227692	Down
hsa-miR-520a-3p	8.42184	5.32156	0.000422	0.0243993	Down
hsa-miR-520 g-5p	5.97107	1.538027	0.000432	0.0243993	Down
hsa-miR-518c-5p	6.77756	2.296079	0.000515	0.0276265	Down
hsa-miR-518f-3p	6.99941	4.125711	0.000617	0.0299196	Down
hsa-miR-519d-5p	6.74128	− 0.16451	0.000636	0.0299196	Down
hsa-miR-520a-5p	7.4342	3.430817	0.000641	0.0299196	Down
hsa-miR-525-3p	6.41012	− 0.41056	0.00072	0.0322016	Down
hsa-miR-520c-5p	7.63178	4.29367	0.000901	0.0329712	Down
hsa-miR-523-5p	7.96623	5.705226	0.001033	0.0329712	Down
hsa-miR-522-5p	7.96623	5.705226	0.001033	0.0329712	Up
hsa-miR-518e-5p	7.96623	5.705226	0.001035	0.0329712	Down

### Validation of novel miRNAs and miRNAs with overlapping expression patterns using qRT-PCR in 15 pairs of HCC samples with or without HBV infection

**Fig. 5:**
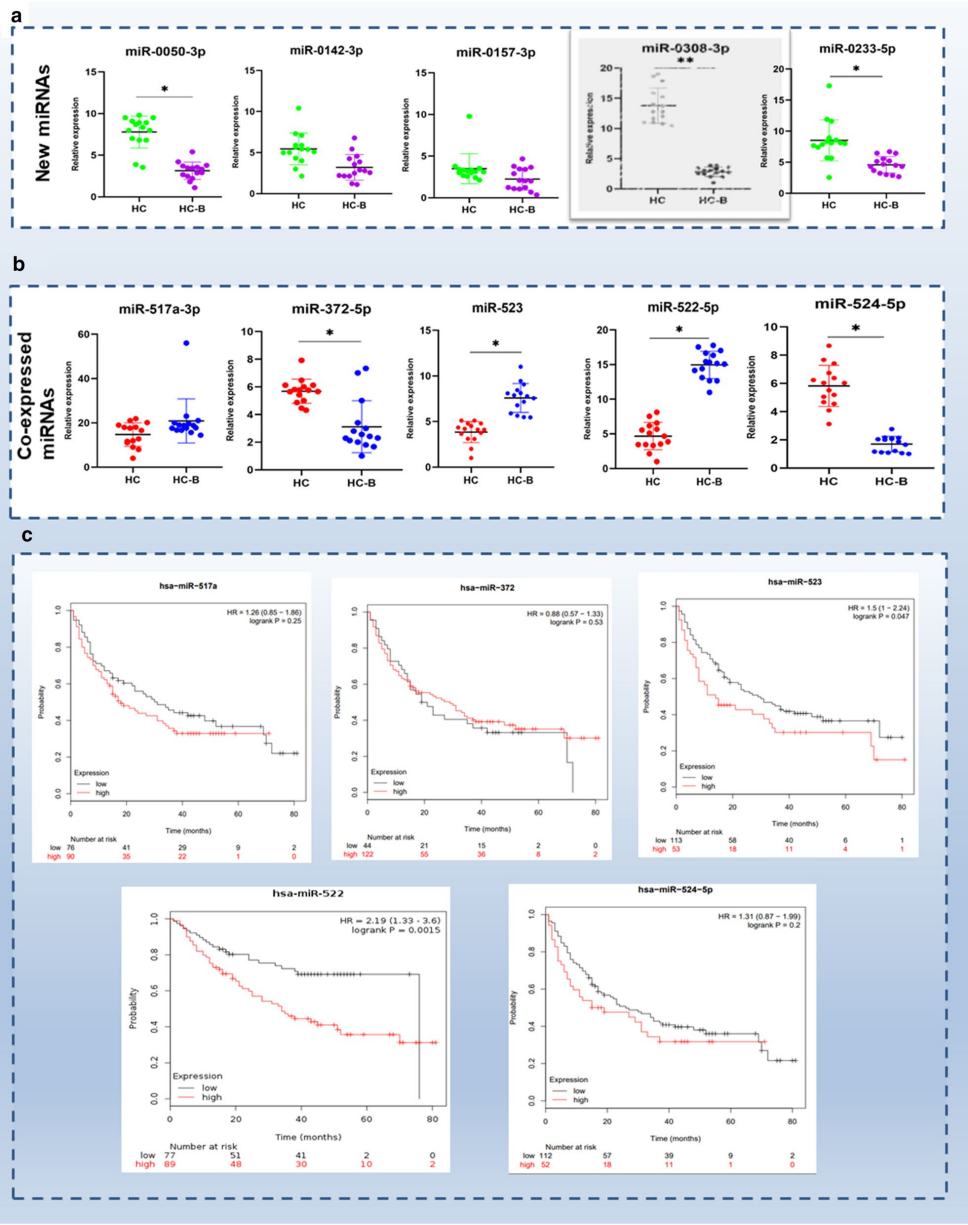
Validation of the miRNAs’ expression via qRT-PCR and a survival analysis based on miRNAs in tissue samples from HBV-positive HCC patients. **a** Novel miRNAs including miR-0050-3p, miR-0142-3p, miR-0157-3p, miR-0308-3p, and miR-0233-5p were selected randomly and validated in 15 pairs of HCC patients with or without HBV infection. **b** miRNAs with overlapping expression patterns, including miR-517a-3p, miR-372-5p, miR-523, miR-522-5p, and miR-524-5p, were selected randomly and validated in 15 pairs of HCC patients with or without HBV infection. **c** Kaplan–Meier analysis of overall survival based on miR-517a, miR-372, miR-523, miR-522, and miR-324-5p in HBV-positive HCC patients from the TCGA database. Patients with high miR-324-5p expression had a significantly poorer long-term prognosis

To evaluate the miRNAseq and TCGA mining results, we conducted qRT-PCR to verify the expression patterns of several miRNAs selected at random. For validation of the novel miRNAs, we selected the following: miR-0050-3p, miR-0142-3p, miR-0157-3p, miR-0308-3p and miR-0233-5p. Compared to the HBV-negative HCC samples, miRN-0050-3p, miR-0233-5p and miR-0308-3p were significantly downregulated, whereas miR-0142-3p and miR-0157-3p were downregulated but this did not reach significance (Fig. 5a).

For validation of the miRNAs with overlapping expression patterns, we selected miR-517a-3p, miR-372-5p, miR-524-5p, miR-523, and miR-522-5p. Compared to the HBV-negative HCC samples, miRN-517a-3p, miR-523 and miR-522-5p were significantly upregulated, whereas miR-372-5p and miR-524-5p were significantly downregulated (Fig. 5b). In Table 4, the miR-517a-3p expression was downregualted in HBV-negative HCC samples whereas upregualted in the validation test. The possible explain may be associated with heterogeneity of the HCC sample which needs further investigation.

To evaluate the clinical significance of these five miRNAs with overlapping expression patterns further, we performed a Kaplan–Meier analysis of overall survival (OS) rates based on the TCGA database data. The results showed that HBV-positive HCC patients with high miR-523, miR-517a, and miR-524 expression had a poorer long-term prognosis, although this finding was significant only for miR-522 (HR 2.19, 95% CI 1.33–3.6, *p* = 0.0015) and miR-523 (HR 1.5, 95% CI 1–2.44, *p* = 0.0047) (Fig. 5c).

### miR-0308-3p expression may play a role in regulation of the HCC cell cycle

**Fig. 6:**
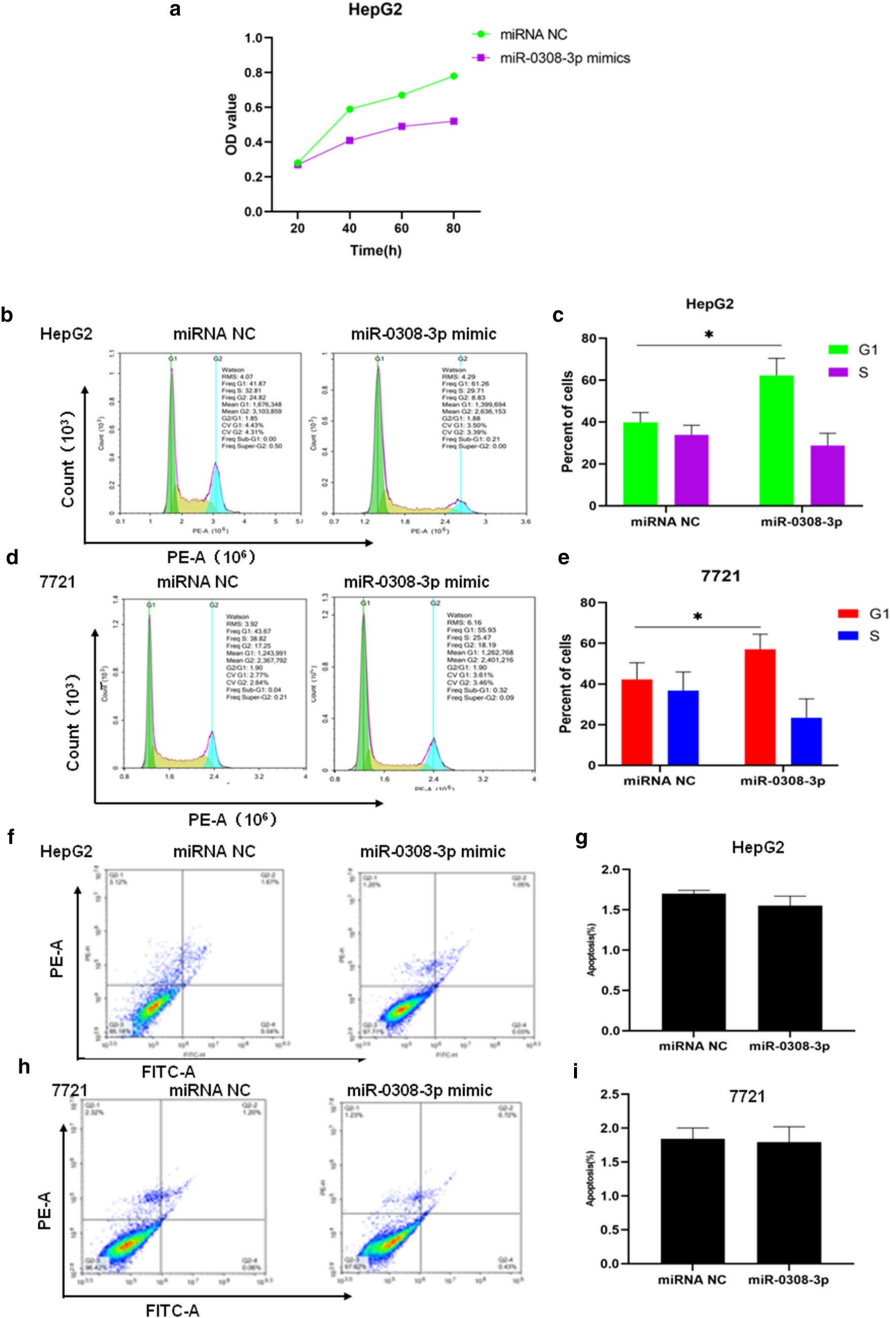
Functional analysis of the newly identified miR-0308 in both HepG2 and 7721 cell lines. **a** HepG2 cells were transfected with 50 nM of miR-0308 mimic or miR-NC and cell proliferation was evaluated via. A CCK-8 assay 48 h after transfection. *p < 0.05 as compared to control miR-NC treated cells. **b** The effects of miR-0308 on the distribution of G_1_ and S phases in cells using flow cytometry. A representative flow cytometry histogram of the cell-cycle progression of HepG2 cells is shown with and without the miR-0308 mimic. **c** Quantitative measurement of the G_1_ and S phases in HepG2 cells with or without the miR-0308 mimic. **d** The effect of miR-0308 on the distribution of G_1_ and S phases in irradiated cells using flow cytometry. A representative flow cytometry histogram of cell cycle progression of 7721 cells is shown with or without the miR-0308 mimic. **e** Quantitative measurement of the G_1_ and S phases of 7721 cells with or without the miR-0308 mimic. **f** Annexin V Flow cytometry assay of apoptosis in HepG2 cells co-transfected with or without the miR-0308 mimic. **g** Quantification of apoptosis, displaying the effects of miR-0303 on induction of apoptosis in HepG2 cells. **h** Annexin V Flow cytometry assay of apoptosis in 7721 cells co-transfected with or without the miR-0308 mimic. **i** Quantification of the apoptosis, displaying the effects of miR-0303 on the induction of apoptosis in 7721 cells. All data are presented as mean ± SD of three independent experiments; *p < 0.05 compared to the control group

To explore the role of novel miRNAs in HBV-positive HCC further, we performed miRNA-expression, cell-proliferation, cell-cycle, and apoptosis assays on HepG2 and SMMC-7721 cell lines. We then selected miR-0308-3p to investigate its effect on HepG2 cell proliferation using the CCK-8 assay. Following transfection with the miR-0308 mimic, HepG2 cell proliferation decreased significantly compared to cells transfected with NC (*p* < 0.05, Fig. 6a). We also investigated the effect of miR-0308-3p on the cell cycles of HepG2 and SMMC-7721 cell lines. Following transfection with the miR-0308 mimic, G1 cell numbers significantly increased in both cell lines compared to cells transfected with NC (*p* < 0.05, Fig. 6b–e).

Next, the effect of miR-0308-3p on cell apoptosis was investigated, but no significant effect was found in either HepG2 or SMMC-7721 (*p* > 0.05, Fig. 6f–i). Together, these results imply that increased miR-0308-3p expression contributes to the activation of G1/S arrest in HCC cancer cells.

### miR-0308-3p downregulates double CDK6/Cyclin D1 expression by directly targeting the CDK6/Cyclin D1 3′-UTR

**Fig. 7:**
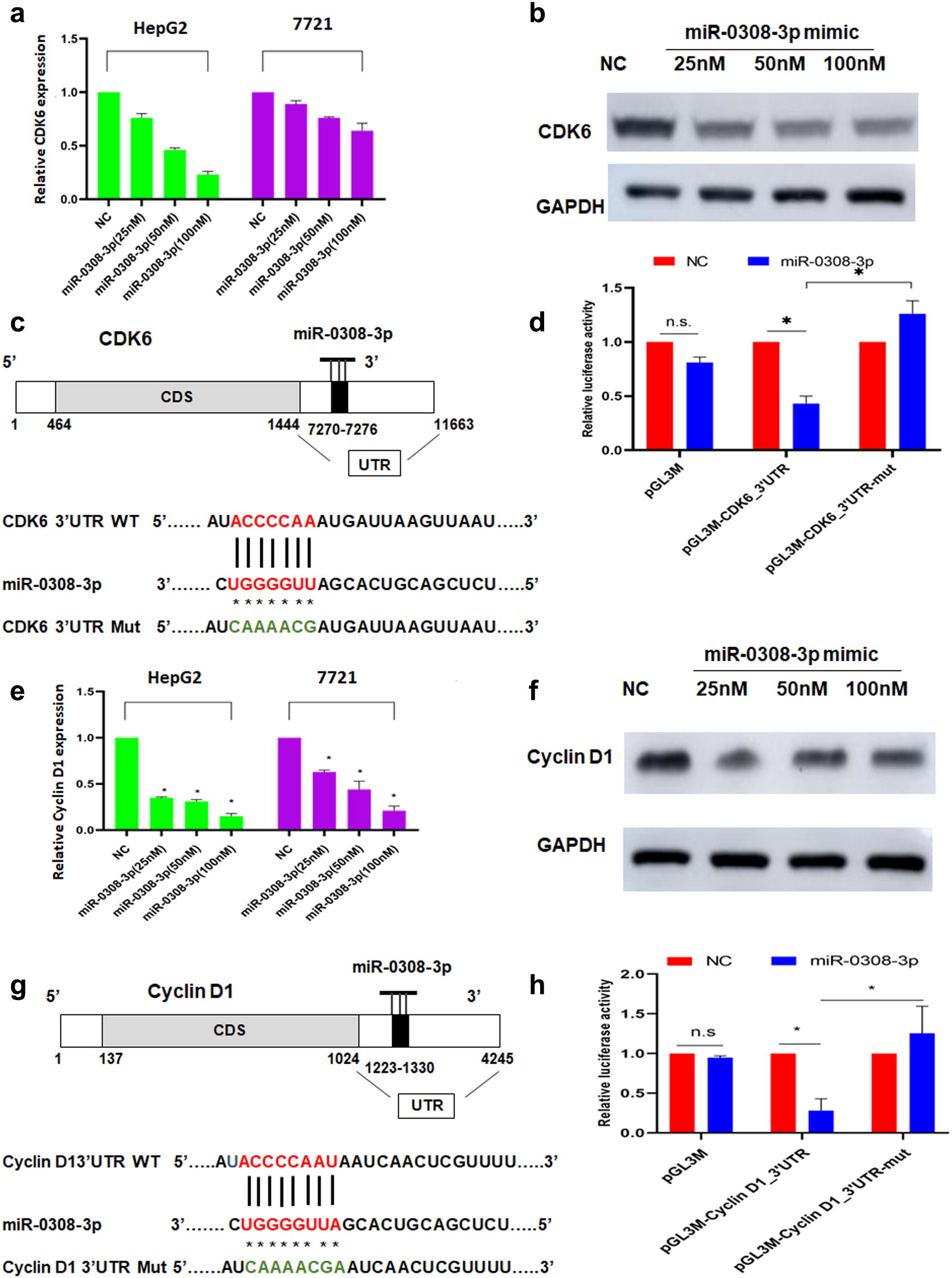
Effects of the miR-0308 mimic on CDK6 and Cyclin D1 proteins expression and the mRNA 3ʹUTR activity of CDK6 and Cyclin D1 genes. **a** qRT-PCR was conducted to detect the relative expression of CDK6 in both HepG2 and 7721 cells. **b** Western blotting analysis of CDK6 protein expression in HepG2 cells after treatment with the miR-0308 mimic for 24 h at 25 nM, 50 nM, and 100 nM, respectively. **c** Predicted target sites of miRNA-0308 in CDK6 3ʹUTR, and the wild-type and mutated CDK6 mRNA 3ʹUTR sequences that were inserted into the pmirGLO plasmids to construct the luciferase reporter vectors. **d** Activity of the luciferase reporter; higher activity indicates miR-1246 interacting with the CDK6 3ʹUTR target sequence. Cells were co-transfected with the reporter vectors and either the miR-0308 mimic or NC. Luciferase activity was analyzed at 48 h after transfection. The reporter assay was repeated three times. **e** qRT-PCR was conducted to detect the relative expression of Cyclin D1 in both HepG2 and 7721 cells. **f** Western blotting analysis of Cyclin D1 protein expression in HepG2 cells after treatment with the miR-0308-3p mimic for 24 h at 25 nM, 50 nM, and 100 nM, respectively. **g** Predicted target sites of miRNA-0308 in Cyclin D1 3’UTR, and the wild-type and mutated Cyclin D1 mRNA 3’UTR sequences that were inserted into the pmirGLO plasmids to construct the luciferase reporter vectors. **h** Activity of the luciferase reporter; higher activity indicates miR-1246 interacting with the Cyclin D1 3’UTR target sequence. Cells were co-transfected with the reporter vectors and either the miR-0308 mimic or NC. Luciferase activity was analyzed at 48 h after transfection. The reporter assay was repeated three times. *p < 0.05; n.s., no significance

To uncover the underlying mechanism of miR-0308-3p in activation of HCC G1/S cell arrest, we performed qRT-PCR and western blot analysis. The results showed that the levels of mRNA and protein expression related to double CDK6/Cyclin D1 decreased gradually as the concentration of the miR-0308 mimic increased from 25 to 100 nM (Fig. 7a, b).

To explore the direct action of miR-0308-3p on the target CDK6 mRNA 3ʹUTR, pmirGLO-CDK6_3ʹUTR_wt and pmirGLO-CDK6_3ʹUTR-Mut reporter vectors were constructed. The sequences of the 3ʹUTR region of CDK6 mRNA containing the miR-0308-3p targeting sequence and its mutant were cloned separately, downstream of the pmirGLO plasmid luciferase gene, to construct the reporter vectors. The predicted miR-0308 targeting site and the sequence of the CDK6 mRNA 3ʹUTR are shown in Fig. 7c. The cells were then co-transfected with these reporter vectors and either miR-0308-3p or miR-NC. miR-0308-3p significantly decreased the luciferase activity of the pmirGLO-CDK6_3ʹUTR reporter, but had no effect on the pmirGLO-CDK6_3ʹUTR mutant (Fig. 7d). This result implies that miR-0308-3p acts directly on the target sequence of the CDK6 mRNA 3ʹUTR to suppress CDK6 expression.

The same method was employed to explore the direct action of miR-0308-3p on the target Cyclin D1 mRNA 3ʹUTR. First pmirGLO-Cyclin D1_3ʹUTR_wt and pmirGLO-Cyclin D1_3ʹUTR-Mut reporter vectors were constructed in the same manner as those for CDK6 (Fig. 7e). Cells were co-transfected with these reporter vectors and either miR-0308-3p or miR-NC, and miR-0308-3p significantly decreased the luciferase activity of the pmirGLO-Cyclin D1_3ʹUTR reporter (Fig. 7f), but had no effect on the pmirGLO-Cyclin D1_3ʹUTR mutant. This result implies that miR-0308-3p acts directly on the target sequence of the Cyclin D1 mRNA 3ʹUTR to suppress Cyclin D1 expression.

To further role out that downregualtion of CDK1 and CYClin D1 by miR-0308-3p could play a important role to mediate miR-0308-3p-induced G1/S cell cycle arrest, the expression vectors for miR-0308-3p-resistance CDK6 and Cyclin D1 vectors were constructed and trnasfected in the HepG2 cells, as Additional file 2 :Figure S1 shown, compared to the control group, G1 cell numbers in cells treated with miR-0308-3p-resistant pEX-3- Cyclin D1 vector or CDK1 vector were significantly rescued (p < 0.05).

## Discussion

Microarray analysis and high-throughput analysis have enabled tens of thousands of miRNAs to be identified over the past few decades [34]. More recently, studies to identify cancer-related miRNAs and predict or evaluate their functions have been increasing rapidly [35]. Several miRNAs have also been found to play essential roles in the carcinogenesis of HCC; [36–39] however the number of miRNAs characterized in HBV-positive HCC is still limited and worth further investigation. In light of this, we used high-throughput sequencing and re-analyzed the RNA-seq data from the TCGA database to predict new and potential miRNAs associated with the carcinogenesis of HBV-related HCC. Notably, we found two miRNAs, miR-522 and miR-523, with overlapping expression patterns in both our HBV-positive HCC samples and the TCGA database. These miRNAs were markedly overexpressed in HBV-positive HCC and were associated with a significantly poorer long-term prognosis (miR-522, HR 2.19, 95% CI 1.33–3.6, *p* = 0.0015; miR-523, HR 1.5, 95% CI 1–2.44, *p* = 0.0047).

Of note, we found a novel miR-0308-3p that was markedly downregulated in HBV-positive HCC samples compared to HBV-negative HCC samples and adjacent normal hepatic tissue. Moreover, we found that miR-0308-3p was downregulated in HepG2 cells and 7721 cancer cells, and that elevated expression of miR-0308-3p inhibited proliferation and promoted G1/S arrest but did not influence the apoptosis of cancer cells. This implies that miR-0308-3p may affect the carcinogenesis of HBV-related HCC through regulating G1/S-related genes. Based on this inference, we used bioinformatics to predict that two G1/S-related genes, CDK6 and Cyclin D1, were downstream targets of miR-0308-3p. Furthermore, a dual luciferase reporter activity assay identified that miR-0308-3p acted directly on the target sequence of the CDK6/Cyclin D1 mRNA 3ʹUTR to suppress CDK6/Cyclin D1 expression.

Many reports have revealed that miRNAs are involved in human carcinogenesis. HBV-positive HCC has been found to be associated with altered expression of miRNAs. Wang et al. investigated the miRNA profiles of 12 pairs of chronic HBV-associated HCC samples using microarray analysis [40] and found seven miRNAs (miR-150, miR-342-3p, miR-663, miR-20b, miR-92a-3p, miR-376c-3p and miR-92b) that were specifically altered in HBV-related HCC. Nielsen et al. screened for alterations in miRNA expression in liver-cancer cell lines post-HBV infection and found that miR-192-5p, miR-194-5p, and miR-215-5p were upregulated, providing further evidence that these miRNAs are associated with the development of HBV-related liver disease [41]. Lou et al. mined the NCBI GEO database for HBV-related HCC RNAseq data and found seven upregulated and nine downregulated differentially expressed miRNAs [42]. Our study integrated high-throughput sequencing and TCGA database mining and found further alterations in miRNA expression in HBV-positive versus HBV-negative HCC. Notably, we identified a series of 34 miRNAs with overlapping expression patterns. miRNAs with overlapping expression patterns such as miR-522, miR-518, miR-519, and miR-520 have previously been reported to be upregulated in HBV-positive HCC [43]. In contrast, our results found miR-522, miR-518, miR-519, and miR-520 to be downregulated in HBV-positive HCC. This may have been due to the heterogeneity and plasticity of clinical samples, or the expression of these miRNAs may be dependent on internal or external factors.

After differentially expressed key miRNAs were identified from clinical HCC samples, we performed GO and KEGG bioinformatic analyses to predict the miRNAs’ potential biological functions and signaling pathways. miRNAs are usually used to conduct OS assays to elucidate their prognostic value in cancer patients. Wang et al. analyzed miR-522 expression in HBV-positive HCC tissue using GO and KEGG analyses and found that regulation of the actin cytoskeleton and pathway in cancer are most enrichment role in HCC carcinogenesis [43]. Shi et al. indicated that miR-522 overexpression in HCC tissue was markedly correlated with a poor prognosis and decreased OS in HCC patients [44]. miR-523 has also been reported to be upregulated in acute myeloid leukemia plasma [45], pituitary adenomas [46], colon cancer [47], and uveal melanom [48], but has not previously been reported to be associated with HCC. In our study, we found miR-523to be upregulated in HBV-positive HCC carcinogenesis. Since miR-523 has not been investigated in HBV-positive HCC tissue previously, our results imply that overexpression of miR-523 may serve as a novel biomarker for an unfavorable prognosis in HBV-positive HCC patients.

Exploring the mechanism underlying the carcinogenesis of HBV is critical in prevention of, and therapeutics for, HBV-positive HCC. In recent years, studies focusing on the roles of miRNAs in HBV-positive HCC have increased. A study by Liu et al. demonstrated that HBV preS2 promotes the transcriptional co-activator with PDZ binding motif (TAZ) through miR-338-3p leading to enhanced HCC proliferation and migration [49]. Shi et al. indicated that miR-200a-3p was downregulated by HBV X protein and promoted liver cancer cell proliferation and invasion [50]. Another study found that miR-384 was downregulated in HBV-related HCC tissue and promoted liver cancer cell proliferation and metastasis by targeting the oncogene pleiotrophin [51]. Meanwhile, miR-34c was shown to target TGFβ-induced factor homeobox 2 and repress cell proliferation in HBV-related HCC [52]. Xiang et al. found that upregulation of miR-499a induced HCC carcinogenesis by targeting MAPK6 [53]. Qin et al. illustrated that miR-30b-5p repressed cell proliferation and arrested the cell cycle of a HCC cell line by targeting USP37 [54]. Lamontagne et al. pointed out in a review that altered regulation of miRNA expression may play a critical role in HBV-related cancer development [55].

In general, alteration of miRNA expression leads to altered expression of its targeted genes, which may influence carcinogenesis. For instance, miR-218 can induce cell-cycle arrest at the G1 phase by targeting the 3ʹ-UTR region of CDK6/Cyclin D1 [56], and overexpression of miR-24 increases the percentage of cells in G1 phase in HepG2 and K562 cancer cells [57]. Cell-cycle deregulation is a common feature of HCC, as cancer cells frequently display unscheduled proliferation resulting from disruptions in the cell cycle, such as G1/S arrest [58]. CDK6/Cyclin D1 genes have been reported to be induced during G1/S transition, and G1/S dysregulation is usually mediated by alterations in the activity of CDK6/Cyclin D1. Although there exists limitations in this study including the evidence for that the miR-0308-3p overexpressing HCC are less aggressive and patients have better survival rate remains further investigated, as well as for such large scale screening of miRNA, the cut off as p < 0.01 may not appropriate, we found a novel miRNA, miR-0308-3p, that inhibited cancer cell proliferation and induced G1/S arrest via targeting double CDK6/Cyclin D1 genes. No miRNAs have been previously reported to suppress G1/S transition in HBV-positive HCC, and our results imply that miR-0308-3p may act as an HBV-positive HCC suppressor.

## Conclusion

In summary, this study demonstrated the effect of miR-0308-3p on HBV-positive HCC. MiR-0308-3p upregulation dramatically suppressed HCC cell proliferation and induced G1/S arrest by directly targeting CDK6/Cyclin D1. These findings reveal a novel molecular mechanism of activation of G1/S arrest in HCC and may prove clinically useful for developing new therapeutic targets for HCC. However, the mechanism underlying HBV regulation of miR-0308-3p expression requires further study, and the effect of miR-0308-3p as well as the other newly identified miRNAs and their targets should be verified with further in vitro and in vivo experiments. Furthermore, the molecular mechanism underlying HBV-positive carcinogenesis of liver cells requires additional future studies. Answering these questions may be of significance for the prevention and treatment of HBV-positive HCC.

## Supplementary information

**Additional file 1: Table S1.** Primers used for qRT-PCR of selected novel miRNAs randomly.

**Additional file 2: Figure S1.** Rescue analysis of the effects of *miR*-*0308*-*3p*-*resistance CDK6 and Cyclin D1on G1/S cell cycle.* (A) The effects of miR-0308-3p-resistance CDK6 on the distribution of G_1_ and S phases in cells using flow cytometry. A representative flow cytometry histogram of the cell-cycle progression of HepG2 cells is shown with and without the miR-0308 resistance CDK6. (B) Quantitative measurement of the G_1_ and S phases in HepG2 cells with or without the miR-0308 resistance CDK6. (C) The effects of miR-0308-3p-resistance Cyclin D1 on the distribution of G_1_ and S phases in cells using flow cytometry. A representative flow cytometry histogram of the cell-cycle progression of HepG2 cells is shown with and without the miR-0308 resistance Cyclin D1. (D) Quantitative measurement of the G_1_ and S phases in HepG2 cells with or without the miR-0308 resistance Cyclin D1.

## Data Availability

Not applicable.
