# Supplementary material for: A novel miR-0308-3p revealed by miRNA-seq of HBV-positive hepatocellular carcinoma suppresses cell proliferation and promotes G1/S arrest by targeting double CDK6/Cyclin D1 genes
Source: Cell Biosci. 2020 Feb 27;10:24. doi: 10.1186/s13578-020-00382-7 (PMC7047384; doi:10.1186/s13578-020-00382-7)
Supplement: Supplementary file 1 — Additional file 1: Table S1. Primers used for qRT-PCR of selected novel miRNAs randomly. [file 13578_2020_382_MOESM1_ESM.docx]

Table S1 Primers used for qRT-PCR of selected novel miRNAs randomly

| Novel miRNA ID | primer | sequence |
| --- | --- | --- |
|  | xxx-m0050-3p-FO | AGTGGCAAGATCGTCTGTGA |
| xxx-m0050-3p | xxx-m0050-3p-RE | CTCTGTCTCTCGTCTTGTTGGTAT |
|  | xxx-m0050-3p-PR | CTCAGGGGTCTGTATGGTTGTAGATAG |
|  |  |  |
|  | xxx-m0142-3p-FO | TGACTTCCGCTTACACTAGGATTAGA |
| xxx-m0142-3p | xxx-m0142-3p-RE | CACTTGCTCTCCACTTGTTGGTAT |
|  | xxx-m0142-3p-PR | ACAAGTTGTCTGTATGGTTGGATAGG |
|  |  |  |
|  | xxx-m0157-3p-FO | AGCTCACTCCCTCTTCGTTTTATAGT |
| xxx-m0157-3p | xxx-m0157-3p-RE | CACTTGCTCTCCACTTGTTGGTAT |
|  | xxx-m0157-3p-PR | CTGAACTCGTCTGTATGGTTGGATAGG |
|  |  |  |
|  | xxx-m0233-5p-FO | TTGGACGCAACACTGTGGTTAT |
| xxx-m0233-5p | xxx-m0233-5p-RE | ATGGAGCCTGGGACGAGAC |
|  | xxx-m0233-5p-PR | TCTATACGTCGGGTCCAGAGCGAACG |
|  |  |  |
|  | xxx-m0233-5p-FO | AACGGACCACTGCACTACAGG |
| xxx-m0244-3p | xxx-m0233-5p-RE | CAGTTGGTCAGCAGTTCTTGGTAT |
|  | xxx-m0233-5p-PR | CTTGGTTGTCTGTATGGTTCGATAGGG |
|  |  |  |
|  | xxx-m0276-3p-FO | CCTCAGTGGTGGAACTTGGAA |
| xxx-m0276-3p | xxx-m0276-3p-RE | CAGTTCCTCAGCAGATGTTGGTAT |
|  | xxx-m0276-3p-PR | CACGGCTTGTCTGTATGGTTGGATAGGG |
|  |  |  |
|  | xxx-m0308-3p-FO | CACGCACTGGGGTAAGCACT |
| xxx-m0308-3p | xxx-m0308-3p-RE | ATGGAGCCTGGGACGAGAC |
|  | xxx-m0308-3p-PR | CAGCTCTGTCGGGTCCAGAGCG |
|  |  |  |
|  | xxx-m0345-3p-FO | ACCCTTCCAGCATTGGACTG |
| xxx-m0345-3p | xxx-m0345-3p-RE | CAGTTCCTCAGCAGATGTTGGTAT |
|  | xxx-m0345-3p-PR | CACCAGTCTGTATGGTTGGATAGGG |
|  |  |  |
|  | xxx-m0363-3p-FO | ATCCCTCGGTCCCTAACCC |
| xxx-m0363-3p | xxx-m0363-3p-RE | CAGTTCCTCAGCAGATGTTGGTAT |
|  | xxx-m0363-3p-PR | CCTCCGGACGTCTGTATGG |
|  |  |  |
|  | xxx-m0482-5p-FO | GGCAGGTGAGAGCTGGATTC |
| xxx-m0482-5p | xxx-m0482-5p-RE | CAGTTCCTCAGCAGATGTTGGTAT |
|  | xxx-m0482-5p-PR | CATGGGGCGTCTGTATGGTTGGATAG |
|  |  |  |
|  | U6 snRNA-FO | CGCTTCGGCAGCACATATAC |
| U6 snRNA | U6 snRNA-RE | TTCACGAATTTGCGTGTCATC |
|  | U6 snRNA-PR-4 | AAGATTAGCATGGCCCCTGCGCA |
