# Supplementary figures and images for: A novel miR-0308-3p revealed by miRNA-seq of HBV-positive hepatocellular carcinoma suppresses cell proliferation and promotes G1/S arrest by targeting double CDK6/Cyclin D1 genes
Source: Cell Biosci. 2020 Feb 27;10:24. doi: 10.1186/s13578-020-00382-7 (PMC7047384; doi:10.1186/s13578-020-00382-7)

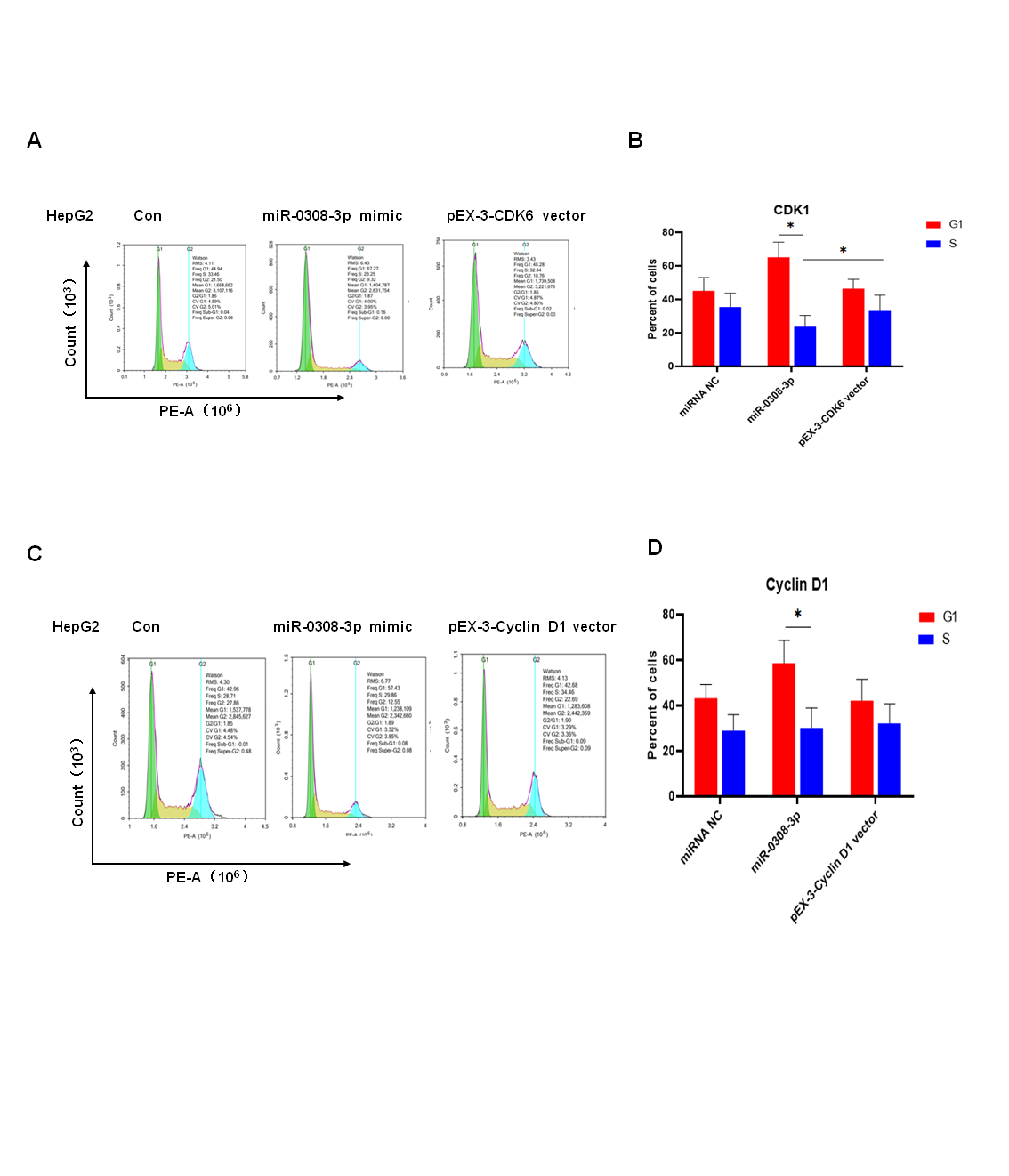

Supplement: Supplementary file 2 — Additional file 2: Figure S1. Rescue analysis of the effects of miR-0308-3p-resistance CDK6 and Cyclin D1on G1/S cell cycle. (A) The effects of miR-0308-3p-resistance CDK6 on the distribution of G1 and S phases in cells using flow cytometry. A representative flow cytometry histogram of the cell-cycle progression of HepG2 cells is shown with and without the miR-0308 resistance CDK6. (B) Quantitative measurement of the G1 and S phases in HepG2 cells with or without the miR-0308 resistance CDK6. (C) The effects of miR-0308-3p-resistance Cyclin D1 on the distribution of G1 and S phases in cells using flow cytometry. A representative flow cytometry histogram of the cell-cycle progression of HepG2 cells is shown with and without the miR-0308 resistance Cyclin D1. (D) Quantitative measurement of the G1 and S phases in HepG2 cells with or without the miR-0308 resistance Cyclin D1. [file 13578_2020_382_MOESM2_ESM.tif]
